# Brown Adipose Tissue in Morbidly Obese Subjects

**DOI:** 10.1371/journal.pone.0017247

**Published:** 2011-02-24

**Authors:** Guy H. E. J. Vijgen, Nicole D. Bouvy, G. J. Jaap Teule, Boudewijn Brans, Patrick Schrauwen, Wouter D. van Marken Lichtenbelt

**Affiliations:** 1 Department of Human Biology, School for Nutrition and Toxicology and Metabolism - NUTRIM, Maastricht University, Maastricht, The Netherlands; 2 Department of General Surgery, Maastricht University Medical Centre, Maastricht, The Netherlands; 3 Department of Nuclear Medicine, Maastricht University Medical Centre, Maastricht, The Netherlands; University of Padova, Medical School, Italy

## Abstract

**Background:**

Cold-stimulated adaptive thermogenesis in brown adipose tissue (BAT) to increase energy expenditure is suggested as a possible therapeutic target for the treatment of obesity. We have recently shown high prevalence of BAT in adult humans, which was inversely related to body mass index (BMI) and body fat percentage (BF%), suggesting that obesity is associated with lower BAT activity. Here, we examined BAT activity in morbidly obese subjects and its role in cold-induced thermogenesis (CIT) after applying a personalized cooling protocol. We hypothesize that morbidly obese subjects show reduced BAT activity upon cold exposure.

**Methods and Findings:**

After applying a personalized cooling protocol for maximal non-shivering conditions, BAT activity was determined using positron-emission tomography and computed tomography (PET-CT). Cold-induced BAT activity was detected in three out of 15 morbidly obese subjects. Combined with results from lean to morbidly obese subjects (n = 39) from previous study, the collective data show a highly significant correlation between BAT activity and body composition (P<0.001), respectively explaining 64% and 60% of the variance in BMI (r = 0.8; P<0.001) and BF% (r = 0.75; P<0.001). Obese individuals demonstrate a blunted CIT combined with low BAT activity. Only in BAT-positive subjects (n = 26) mean energy expenditure was increased significantly upon cold exposure (51.5±6.7 J/s versus 44.0±5.1 J/s, P = 0.001), and the increase was significantly higher compared to BAT-negative subjects (+15.5±8.9% versus +3.6±8.9%, P = 0.001), indicating a role for BAT in CIT in humans.

**Conclusions:**

This study shows that in an extremely large range of body compositions, BAT activity is highly correlated with BMI and BF%. BAT-positive subjects showed higher CIT, indicating that BAT is also in humans involved in adaptive thermogenesis. Increasing BAT activity could be a therapeutic target in (morbid) obesity.

## Introduction

Brown adipose tissue (BAT) is known for its capacity to generate heat in response to cold or diet to maintain thermal balance. The regulated production of heat is called adaptive thermogenesis. BAT is the main tissue for this adaptive thermogenesis in rodents and most likely in human infants.[1], [2] Although several early anatomical studies suggested that brown adipose tissue is present in adult humans [3], [4], [5], its physiologic relevance was believed to be marginal for most.[5], [6] However, recent prospective, controlled studies showed that functional BAT is detectable in lean and obese adult humans after exposure to mild cold.[7], [8], [9] We showed a high incidence of cold induced BAT activity that was inversely related to body mass index (BMI) and body fat percentage (BF%).[7] Other studies had similar findings.[8], [9], [10]

Cold-stimulated adaptive thermogenesis in BAT to increase energy expenditure is suggested as a possible therapeutic target for the treatment of obesity.[11], [12] Cold can indeed stimulate adaptive thermogenesis, but differences in body composition may be correlated with the magnitude and type of response. We showed that lean subjects increase energy expenditure significantly in response to mild cold, whereas obese subjects have a blunted cold-induced thermogenesis (CIT) and show a larger insulative response.[13] If BAT is directly responsible for CIT, the absence of CIT in obesity suggests that BAT is reduced or absent. In addition, if the presence of BAT is structurally lower in obesity, this could be a risk factor for the development of obesity. Not being able to burn off excess calories leads to a positive energy balance and predisposes to develop obesity. This could explain the differences in weight gain in classic overfeeding studies.[14]

In our previous report BAT abundance in obese subjects was relatively low, though detectable in most individuals. In the current study we address BAT activity in morbid obesity, a severe form of obesity characterized by a BMI ≥35 kg/m^2^ with concomitant disease or ≥40 kg/m^2^ without.

To ensure maximal non-shivering thermogenesis (NST), as occurs before the onset of shivering,[15] every subject was exposed to an individualized cooling protocol. We hypothesized that morbidly obese in comparison to lean subjects show reduced BAT activity upon cold exposure. Furthermore, we examined the role of BAT activity in cold-induced thermogenesis in these morbidly obese subjects.

## Methods

The study was reviewed and approved by the medical ethics committee of the Maastricht University Medical Centre. Written informed consent was obtained from fifteen morbidly obese subjects, two male and thirteen female. The mean BMI was 42.1±3.8 kg/m^2^ (range 34.8–48.3 kg/m^2^), mean body fat percentage 48.5±4.5% (range 37.9–54.7%), mean weight 123.9±16.7 kg (range 98.2–155.0 kg) and mean age 39.2±8.1 years (range 24–51 years). Subjects were not included when diagnosed with diabetes or use of beta-blockers. One female subject used levothyroxin for hypothyroidism and was euthyroid for several years. One female subject used azathioprine and mesalazine for Crohn's disease. They were studied in the morning from 8 a.m. to 1 p.m. in a fasted state; only water consumption was allowed after 10 p.m. the night before measurements. During the measurements the subjects wore light standardized clothing (socks 0.02 clo, shirt 0.09 clo, sweatpants 0.28 clo, underwear 0.04 clo, total clo factor 0.43 clo).

Core temperature was measured by a telemetric pill (CoreTemp, USA). This measurement failed in two subjects. Skin temperature was measured by wireless iButtons at the 14 ISO-defined skin sites.[16] Subjects were placed in a specially equipped air permeable tent (Colorado altitude training, USA), which functioned as a transportable climate room at the department of nuclear medicine. The tent was cooled by an air-conditioning, which can be controlled to maintain the temperature inside the tent with an accuracy of 1°C [Kingma et al., submitted]. Subjects were placed in a semi-supine position in a nephrodialysis chair to lay comfortable during the personalized cooling protocol. Previous studies in our group showed high NST in lean subjects at the fixed temperature of 16°C, without shivering.[17] However, in the current study group pilot experiments showed a lower onset temperature of shivering, with high interindividual variation. Therefore we used a personal cooling protocol to ensure maximal NST in the morbidly obese state. The level of cooling (temperature) was attuned to each individual, i.e. close to the cold level that induces shivering. This protocol was performed with subsequent FDG-PET-CT-imaging, because the intraindividual response to cold exposure shows high variation.[17] In order to cool the dorsal site of the body, a water perfused cooling mattress was used (cooling device; Blankett roll, Cincinatti sub zero 2000, USA). Energy expenditure was measured for three hours by indirect calorimetry (Oxycon, Jaeger, Germany). One hour in thermoneutral conditions was followed by two hours of personalized cooling. In the first hour the room temperature was 22.3±0.4°C (baseline), followed by the second hour where the temperature of the room and cooling mattress were decreased until subjects subjectively reported shivering. This was confirmed by continuous measurement of muscle activity (pectoralis major) by use of on-skin electrodes attached to an electromyograph (Nicolet Viking, Nicolet Biomedical Inc, USA). When shivering occurred, the air and water temperatures were increased by steps of 1°C until shivering just stopped. In this manner NST was maximized for each individual without shivering. Stable cooling conditions were reached within 28.6±11.8 minutes. After one hour of cold exposure 74 MBq of ^18^-Fluoro-Deoxy-Glucose (FDG) was injected. Cold exposure was maintained for another hour. To exclude the artifact of muscle activity, subjects were instructed to lay still. After this hour subjects were transferred to the positron emission tomography and computed tomography scanner (PET-CT-scanner) (Gemini TF PET-CT, Philips, the Netherlands). The scanning protocol and data interpretation were identical to our previous study protocol.[7] Energy expenditure, room temperatures and body temperatures were sampled on a one minute basis. On a separate occasion body composition was measured with a dual x-ray absorptiometry (DXA, Hologic, type Discovery A, USA). Statistical analysis was performed with PASW Statistics 18.0 for Mac OS X 10.6.4. Reported data is expressed as means ±SD. Total BAT activity is expressed in kiloBequerel (kBq). To compare findings before and after cold exposure paired t-tests were used; unpaired t-tests were used to compare BAT-positive to BAT-negative subjects. To identify correlations between variables linear regression analyses were conducted. For the relation between brown-adipose-tissue activity and body composition, exponential curve fitting was applied.

## Results

### Individual cooling conditions

Temperature conditions before the onset of shivering were variable. Stable cooling temperatures were established in fourteen subjects (room temperature 14.7±2.4°C, inlet temperature of cooling mattress 13.3±4.7°C). One female subject (BMI 38.7 kg/m^2^, body fat 49.9%, age 44 years, BAT 0 kBq) did not report shivering subjectively nor electromyographically at the lowest possible room (12°C) and cooling mattress (inlet temperature of water mattress 4°C) conditions.

### BAT activity

**Figure 1:**
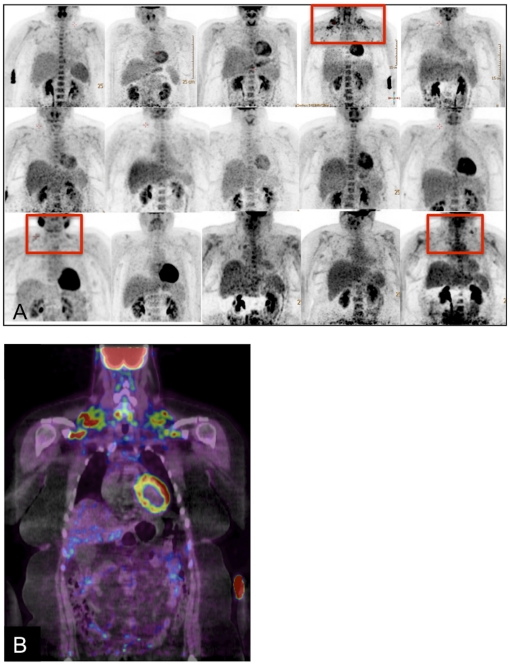
PET-images of fifteen morbidly obese subjects. Three female subjects showed BAT activity in the supraclavicular area, highlighted by a red rectangle (A). Combined PET- and CT-imaging shows FDG-uptake in supraclavicular adipose tissue (this was the morbidly obese subject that showed the most BAT activity) (B).

In the current morbidly obese group mean BAT activity was low (5.5±14.8 kBq, range 0.0–54.5 kBq). Only three female subjects showed a cold induced increased FDG uptake in the neck and supraclavicular area on FDG-PET-CT-imaging (Figure 1). In these three subjects, the BAT activity was 4.48 kBq (BMI 42.5 kg/m^2^, body fat 51.2%, age 47 years), 23.61 kBq (BMI 36.9 kg/m^2^, body fat 50.8%, age 47 years) and 54.45 kBq (BMI 46.3 kg/m^2^, body fat 53.7%, age 32 years). No BAT activity was detected in the other 12 subjects.

### Energy expenditure

On average, energy expenditure during cooling compared to baseline did not increase significantly (baseline 41.9±3.3 J/s, mild cold 43.7±4.8 J/s, P = 0.100) (Table 1). However, a large interindividual variation was evident. The calorimetry measurement in one female subject failed and was excluded from analysis.

**Table 1:** Energy expenditure, body core temperature, mean skin temperature and core-skin temperature gradient in thermoneutral conditions (TN) and during mild cold (Cold) in morbidly obese subjects.

	TN	Min	Max	Cold	Min	Max	P value
Energy expenditure (J/s)	41.9±3.3	37.9	49.6	43.7±4.8	35.7	51.1	0.100
Body core temperature (°C)	37.2±0.4	36.5	37.8	37.5±0.3	36.9	37.9	0.048
Mean skin temperature (°C)	31.7±0.6	30.4	32.9	27.7±1.5	25.2	29.6	<0.001
Core-mean skin gradient (°C)	5.6±0.6	4.9	6.7	9.9±1.6	7.3	12.2	<0.001

### Core and skin temperature

During cooling we observed a significant rise in core temperature (37.2±0.4°C in thermoneutral conditions (TN) versus 37.5±0.3°C during cold exposure (T_Cold_), P = 0.048) and a drop in mean skin temperature (TN 31.7±0.6°C versus T_Cold_ 27.7±1.5°C, P<0.001) (Table 1). Compared to thermoneutral conditions, cooling significantly increased the core-mean skin temperature gradient (TN 5.6±0.6°C versus T_Cold_ 9.9±1.6°C, P<0.001) (Table 1).

### Relationship of BAT - BMI

**Figure 2:**
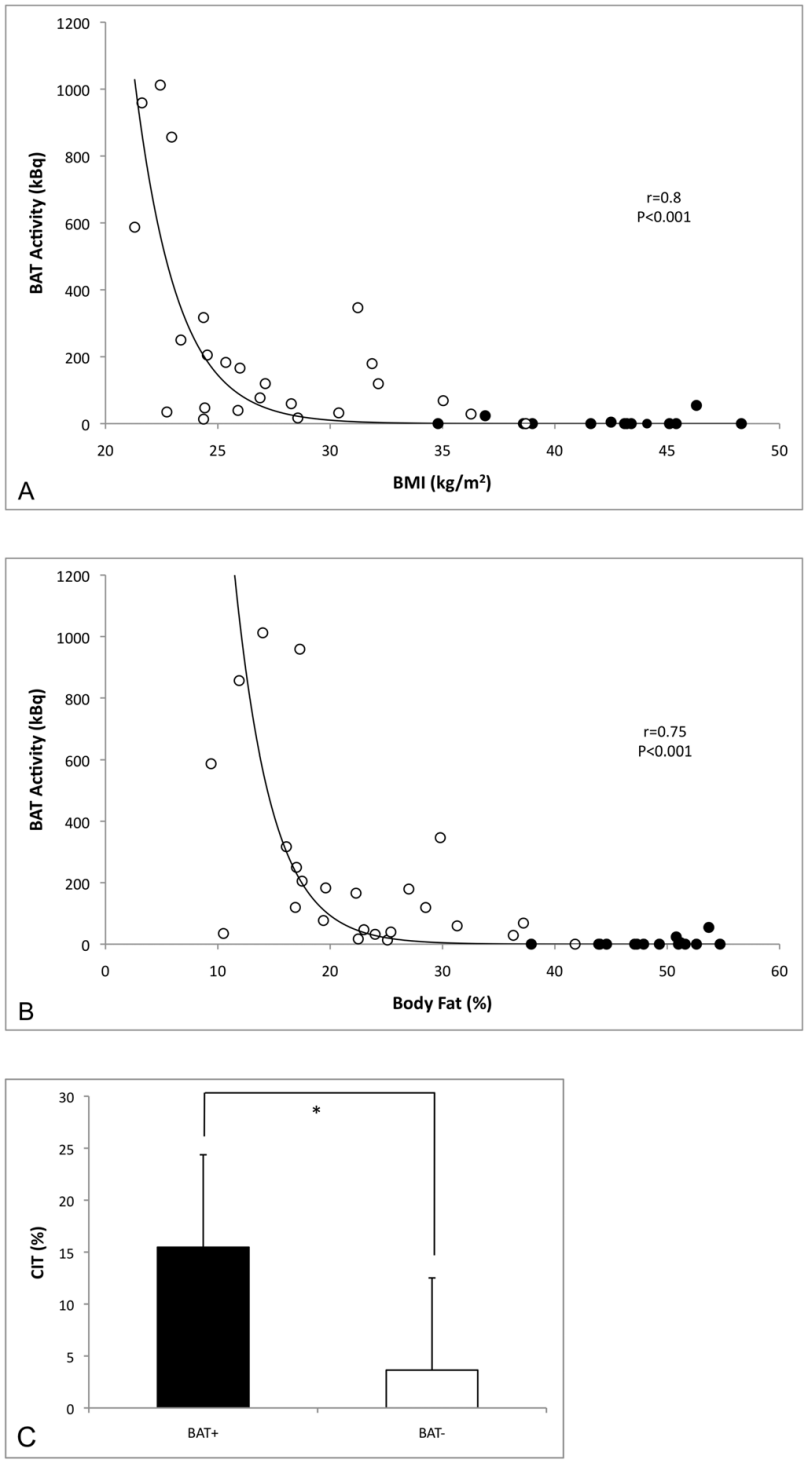
Brown adipose tissue activity in relation to body mass index (A) and body fat percentage (B). The black dots indicate the current study group, the open dots indicate previously performed measurements. Cold-induced thermogenesis (CIT), denoted in percentages, is significantly increased in 26 BAT-positive (BAT+) compared to 13 BAT-negative (BAT-) subjects (C). *: P<0.05.

When combining the data from the current and our previous study,[7] we found strong correlations between BAT activity and BMI (r = 0.80, P<0.001), BAT activity and body fat mass (r = 0.80, P<0.001), and BAT activity and BF% (r = 0.75, P<0.001) (Figure 2).

In the combined group there were 26 subjects that showed active BAT on cold exposure, whereas 13 subjects did not. We therefore reanalysed our energy expenditure data in BAT-positive and BAT-negative subjects (age: 26.1±7.1 yrs, range 18–47 yrs versus 37.1±9.4 yrs, range 20–51 yrs, P = 0.001, and body fat %: 25.9±11.9% versus 47.2±4.7%, P<0.001 in BAT+ vs BAT- resp.). There was no difference in fat free mass, an important contributor to resting metabolic rate (RMR), between both groups (68.6±9.9 kg versus 64.2±9.8 kg, P = 0.195). RMR under thermoneutral conditions did not differ between BAT-positive and BAT-negative subjects (44.6±5.2 J/s versus 42.4±3.3 J/s, P = 0.123), but after cold exposure BAT-positive subjects showed a higher energy expenditure (51.5±6.7 J/s versus 44.0±5.1 J/s, P = 0.001). Very interestingly, mean energy expenditure after cold exposure increased significantly within the BAT-positive group, but not within the BAT-negative group (+15.5±8.9% versus +3.6±8.9%, P = 0.001, Figure 2C).

In the BAT-negative group the core temperature was higher, both in thermoneutral conditions (37.1±0.4°C versus 36.7±0.4°C, P = 0.018) and after cold exposure (37.4±0.4°C versus 36.9±0.3°C, P = 0.001). However, mean skin temperatures did not show a significant difference in thermoneutral conditions (31.9±0.6°C versus 32.3±0.7°C, P = 0.076), nor during cold exposure (28.0±1.2°C versus 28.6±1.2°C, P = 0.135). The gradient between core temperature and mean skin temperature was larger in the BAT-negative group in thermoneutral conditions (5.3±0.5°C versus 4.5±0.8°C, P = 0.004) and during cold exposure (9.6±1.5°C versus 8.3±1.4°C, P = 0.023).

## Discussion

In this study active BAT was detected in only three out of fifteen morbidly obese patients, suggesting that morbid obesity is associated with low BAT activity. On the other hand, the data also demonstrate that even in this group BAT is present in some subjects and can be activated by cold exposure. Nevertheless, the combined results of morbidly obese subjects with those of previously reported lean and obese subjects, clearly show that body composition is highly related to BAT activity. This is in line with lower UCP-1-presence in the intraperitoneal adipose tissue of morbidly obese compared to lean subjects.[18]

A limitation in our analysis is fact that our previous study was composed of male subjects, whereas the morbidly obese subjects were mostly females. However, retrospective studies have actually shown higher BAT activity in women,[19], [20] which would only further strengthen the conclusion that BAT activity is reduced in obesity.

The obese subjects generally show a high insulative response. Therefore, it is important to note that we used an individualized cold exposure protocol that achieves maximal non-shivering conditions. Despite this approach, the major part of obese and morbidly obese subjects did not show BAT activation. In the three BAT-positive morbidly obese subjects, two subjects showed pronounced CIT (BAT 23.61 kBq; CIT 18.76%, BAT 4.48 kBq; CIT 6.04%). Unfortunately, in the third subject (BAT 54.45 kBq) measurement of energy expenditure failed for technical reasons. However, CIT in the two BAT positive morbidly obese subjects was high compared to the average of the morbidly obese group (mean CIT 4.45±9.33%). This suggests a possible relation between active BAT after cold exposure and CIT. Since approximately two-thirds of the total group of subjects showed cold-induced BAT activity whereas one-third did not, we further tested the hypothesis that BAT-positive subjects would have higher (cold-induced) thermogenesis. Interestingly, subjects that have active BAT also have a significant increase in energy expenditure during cold exposure, whereas cold exposure did not increase energy expenditure in BAT-negative subjects. Although age is significantly different in BAT-positive and BAT-negative subjects, the range between both groups (18–47 yrs versus 20–51 yrs) strongly overlaps, with active and inactive BAT spread over all age quartiles.

After we had previously shown that BAT activity was related to RMR, the additional analysis in this study shows that BAT activity may in fact be involved in adaptive thermogenesis. The role of FFM in this process can be excluded, since there is no significant difference in FFM between the analyzed groups. Therefore, the data suggest that - like in rodents - BAT is indeed involved in cold-induced thermogenesis in humans. This is in line with earlier findings in small study groups.[10]

The main native BAT depot is located supraclavicular.[3] That depot shows FDG-uptake after cold exposure in all subjects with active BAT. Biopsies taken from this region show typical BAT cells, mostly surrounded by white fat cells.[8], [21] A pure BAT depot, as seen in rodents, is rare in adult man. Next to native BAT, recently ‘brite’ or ‘beige’ cells derived from white adipose tissue were reported.[22], [23], [24], [25] These cells stem from a different cell lineage than native BAT, but they possess the ability to generate heat by mitochondrial uncoupling similar to native BAT cells.

Most morbidly obese subjects in this study had no detectable BAT activity after cold exposure. From our study, it cannot be concluded that the lack of BAT is cause or consequence of severe obesity in our subjects. However, when the amount of native BAT is minimal or absent, this could lead to severe overweight. For example, mice that lack UCP-1, essential for mitochondrial uncoupling in BAT, become obese when studied under thermoneutral conditions.[26] With a congenital shortage of native BAT, brite cells could therefore be of great therapeutic interest.

On the other hand, in adult man a high amount of thermogenic potential can be present throughout the body, but may remain dormant when insulation makes a metabolic response unnecessary, as may be the case in the morbidly obese state. Indeed, prolonged cold exposure in rodents and rhesus monkeys increases the amount of (brite) BAT, supporting the idea of recruitable BAT.[1] It would therefore be interesting to test if BAT can be recruited in morbidly obese subjects. Next to prolonged cold exposure, other therapeutic targets could recruit BAT: a) weight reduction decreases the insulative capacity that blunts CIT, b) pharmacological intervention, such as sympathicomimetics has a high potential and c) implantation of cultured brown adipocytes could all counteract severe overweight.[12], [27]

In conclusion, this study shows that in an extremely large range of body compositions, BAT activity is highly correlated with BMI and BF%. Active BAT was seen in three out of 15 morbidly obese subjects. BAT-positive subjects showed higher CIT, indicating that BAT is also in humans involved in adaptive thermogenesis.
